# Artificial Intelligence (AI)-Driven Approaches to Manage Postoperative Pain, Anxiety, and Psychological Outcomes in Surgical Patients: A Systematic Review

**DOI:** 10.7759/cureus.84226

**Published:** 2025-05-16

**Authors:** Sachin Agrawal, Rana Veer Samara Sihman Bharattej Rupavath, Priji Prasad Jalaja, Azhar Ushmani, Aashish Mishra, Naga Venkata Satish Babu Bodapati

**Affiliations:** 1 Department of Information Technology, Synechron, Charlotte, USA; 2 Department of Business Administration, National Louis University, Tampa, USA; 3 Department of Surgery, Emory University, Atlanta, USA; 4 Department of Information Security, Amazon Web Service (AWS), Dallas, USA; 5 Department of Computer and Information Science, Eastern Kentucky University, Richmond, USA; 6 Department of Psychiatry and Behavioral Sciences, Sunshine Behavioral Health Services Inc, Bakersfield, USA

**Keywords:** anxiety, artificial intelligence, perioperative care, postoperative pain, psychological outcomes

## Abstract

Postoperative pain, anxiety, and psychological distress significantly impact surgical recovery, yet conventional management strategies often lack personalization. Artificial intelligence (AI) has emerged as a transformative tool in perioperative care, offering potential solutions through predictive analytics, real-time monitoring, and tailored interventions. This systematic review synthesizes evidence on AI-driven approaches for improving postoperative pain, anxiety, and psychological outcomes in surgical patients. Following Preferred Reporting Items for Systematic Reviews and Meta-Analyses (PRISMA) guidelines, we conducted a comprehensive search of PubMed, Institute of Electrical and Electronics Engineers (IEEE) Xplore, Scopus, Web of Science, and Cochrane Library up to April 2025. Ten studies met the inclusion criteria. Risk of bias was assessed using the revised Cochrane risk of bias tool for randomized trials (ROB 2) and Risk Of Bias In Non-randomized Studies-of Interventions (ROBINS-I) for non-randomized studies. Data were narratively synthesized by AI applications (e.g., nociception monitoring, robotics, machine learning (ML)) and outcomes (pain, anxiety, psychological metrics). AI interventions demonstrated efficacy in reducing postoperative pain (e.g., nociception level (NOL)-guided analgesia lowered pain scores by 33% vs. standard care) and anxiety (e.g., interactive robots reduced pediatric preoperative anxiety). ML models predicted pain severity (area under the curve (AUC) up to 0.75) and complications (AUC 0.84) but showed lower accuracy for readmissions (AUC 0.66). Automated psychological interventions reduced opioid use by 36.5%. Limitations included small sample sizes (12 to 201 participants), heterogeneity in AI methods, and short follow-up durations. AI shows promise in personalizing perioperative care, particularly for pain and anxiety management, though standardization and larger trials are needed. Future research should prioritize robust validation, long-term outcomes, and integration into clinical workflows to translate AI’s potential into routine practice.

## Introduction and background

The perioperative period presents a complex interplay of physical and psychological stressors that significantly influence patient outcomes [1]. Postoperative pain, anxiety, and psychological disturbances such as depression, delirium, and post-traumatic stress symptoms remain prevalent among surgical patients, contributing to delayed recovery, increased morbidity, prolonged hospitalization, and reduced quality of life [2]. Conventional approaches to managing these outcomes often rely on standardized protocols and subjective assessments, which may fail to capture the dynamic, individualized nature of patients’ experiences. This underscores the need for more precise, predictive, and personalized strategies in perioperative care [3].

In recent years, artificial intelligence (AI) has emerged as a transformative tool in medicine, offering innovative solutions to complex clinical problems [4, 5]. AI-driven approaches, ranging from machine learning (ML) algorithms and natural language processing to predictive analytics and computer vision, are increasingly being integrated into perioperative workflows [6]. These technologies have demonstrated potential in enhancing clinical decision-making, identifying high-risk patients, and tailoring interventions based on real-time data. Specifically, AI applications in postoperative settings have shown promise in anticipating pain trajectories, evaluating psychological risk profiles, and supporting the timely deployment of targeted therapies [7, 8].

Despite the growing body of literature exploring AI in surgical care [9], there remains a gap in consolidating evidence regarding its role in addressing the intertwined challenges of postoperative pain, anxiety, and psychological outcomes. A systematic synthesis of current research is crucial to understand the scope, effectiveness, and limitations of AI interventions in this domain. By critically evaluating existing studies, this review aims to elucidate how AI is being utilized to predict, monitor, and manage postoperative pain and psychological sequelae, ultimately offering insights into its clinical utility and informing future directions for research and implementation in surgical care.

## Review

Methodology

Eligibility Criteria

This systematic review was conducted following the Preferred Reporting Items for Systematic Reviews and Meta-Analyses (PRISMA) guidelines [10]. Studies were eligible for inclusion if they explored the application of AI in assessing, predicting, or managing postoperative pain, anxiety, or psychological outcomes in surgical patients. We included original peer-reviewed studies involving human subjects, irrespective of the type of surgery or AI methodology used, provided they addressed postoperative psychological or pain-related endpoints. Eligible studies included randomized controlled trials (RCTs), cohort studies, case-control studies, systematic reviews, and cross-sectional analyses. Only articles published in English were considered. Scoping reviews, editorials, conference abstracts, animal studies, and studies without a focus on AI-driven approaches were excluded.

Information Sources

We conducted a comprehensive search of the following electronic databases: PubMed, Institute of Electrical and Electronics Engineers (IEEE) Xplore, Scopus, Web of Science, and the Cochrane Library. The search covered all articles published from inception to April 2025. To ensure the completeness of our findings, we also manually screened the reference lists of included articles and relevant review papers.

Search Strategy

The search strategy was developed in consultation with an academic librarian and tailored to each database using controlled vocabulary (e.g., Medical Subject Headings (MeSH) terms) and free-text terms. The key concepts included terms related to artificial intelligence (e.g., “artificial intelligence,” “machine learning,” “deep learning”), postoperative outcomes (e.g., “postoperative pain,” “postoperative anxiety,” “psychological distress,” “depression,” “mental health”), and surgical patients. Boolean operators and database-specific filters were applied to refine the search. A detailed search strategy for each database is given in Table 1.

**Table 1:** Search strings for five different databases MEDLINE: Medical Literature Analysis and Retrieval System Online; IEEE: Institute of Electrical and Electronics Engineers

Serial number	Database	Search strategy
1	PubMed (MEDLINE)	("artificial intelligence" OR "machine learning" OR "deep learning") AND (surgery OR postoperative) AND (pain OR anxiety OR depression OR "psychological outcomes")
2	IEEE Xplore	("artificial intelligence" OR "machine learning" OR "deep learning") AND (surgery OR postoperative) AND (pain OR anxiety OR depression OR "mental health")
3	Scopus	TITLE-ABS-KEY("artificial intelligence" OR "machine learning" OR "deep learning") AND TITLE-ABS-KEY(surgery OR postoperative) AND TITLE-ABS-KEY(pain OR anxiety OR depression)
4	Web of Science	TS=("artificial intelligence" OR "machine learning" OR "deep learning") AND TS=(surgery OR postoperative) AND TS=(pain OR anxiety OR depression)
5	Cochrane Library	("artificial intelligence" OR "machine learning" OR "deep learning") AND (surgery OR postoperative) AND (pain OR anxiety OR depression)

Selection Process

All retrieved records were imported into EndNote (Clarivate, Philadelphia, PA) for reference management and deduplication. Two independent reviewers (SA and PPJ), from the list of authors, screened the titles and abstracts of all studies for eligibility. Full-text articles of potentially relevant studies were then assessed independently against the inclusion and exclusion criteria. Any disagreements during the screening or eligibility assessment were resolved through discussion or consultation with a third reviewer (AU), who served as a tiebreaker. The selection process was documented and presented using a PRISMA flow diagram.

Data Collection Process

Data were extracted independently by two reviewers using a standardized and pre-piloted data extraction form. Extracted data included study characteristics (author, year, country, design), population details (sample size, demographics, type of surgery), AI methodology (type of algorithm, data input features, training/validation methods), outcome measures (pain scores, anxiety scales, psychological evaluation tools), and key findings. Any discrepancies in data extraction were resolved by consensus or adjudication by a third reviewer.

Data Items

The main data items collected included the type and application of AI model, type of surgical procedure, postoperative psychological or pain-related outcomes, comparison methods (if any), and study conclusions. Secondary data items included funding sources, ethical approvals, and any reported limitations or biases acknowledged by the authors.

Risk of Bias Assessment

Risk of bias for the included studies was assessed using appropriate validated tools based on study design. The revised Cochrane risk of bias tool for randomized trials (ROB 2) [11] was used to evaluate seven studies that used RCT methodology. This tool considers five key domains: the randomization process, deviations from intended interventions, missing outcome data, measurement of the outcome, and selection of the reported result. Each study was assessed independently across these domains and assigned an overall risk of bias rating as “low,” “some concerns,” or “high.”

For the three non-randomized studies (two systematic reviews and one prospective cohort study), the Risk Of Bias In Non-randomized Studies-of Interventions (ROBINS-I) tool [12] was used to ensure methodological rigor. Although ROB is typically recommended for systematic reviews, ROBINS-I was used uniformly to maintain a consistent evaluation framework across non-RCT designs as per reviewer guidance. The ROBINS-I tool evaluates seven domains, including bias due to confounding, participant selection, and outcome measurement, allowing a comprehensive appraisal of the internal validity of non-randomized evidence.

Synthesis Methods

Due to anticipated variability in AI methodologies, outcome measures, and study designs, a narrative synthesis approach was adopted. We grouped studies according to the type of AI model employed (e.g., supervised learning, deep learning), target outcome (e.g., pain, anxiety, depression), and clinical setting.

Results

Study Selection Process

**Figure 1:**
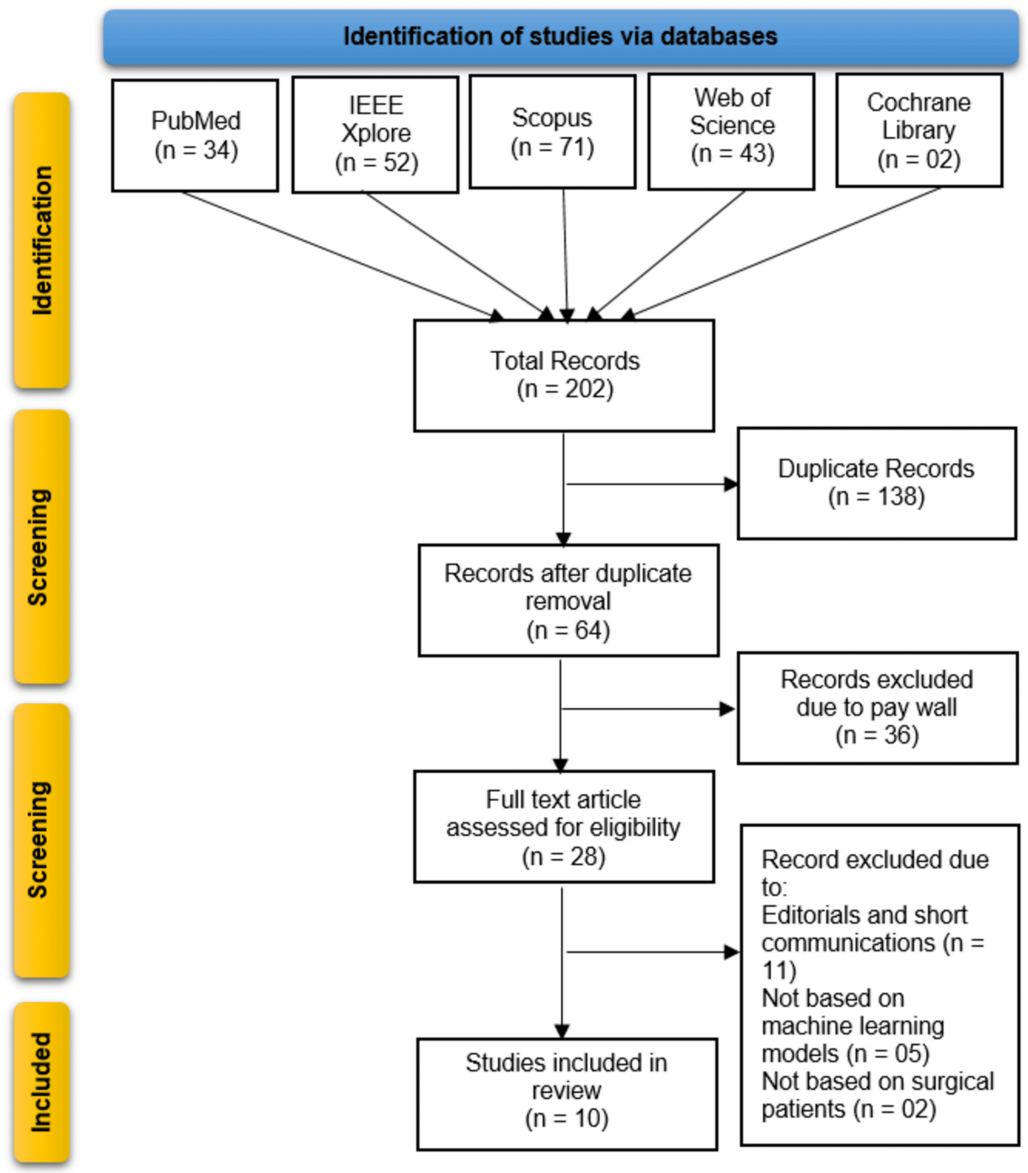
PRISMA flowchart outlining the study selection process PRISMA: Preferred Reporting Items for Systematic Reviews and Meta-Analyses; IEEE: Institute of Electrical and Electronics Engineers

The study selection process followed PRISMA guidelines, beginning with the identification of 202 records from five databases: the Cochrane Library (n = 2), Web of Science (n = 43), Scopus (n = 71), PubMed (n = 34), and IEEE Xplore (n = 52). After removing 138 duplicate records, 64 studies underwent title and abstract screening. Of these, 36 records were excluded due to paywall restrictions, leaving 28 full-text articles assessed for eligibility. Further exclusions were made for editorials/short communications (n = 11), non-ML-based studies (n = 5), and studies not involving surgical patients (n = 2). Ultimately, 10 studies met all inclusion criteria and were incorporated into the systematic review (Figure 1).

Characteristics of the Included Studies

The systematic review included 10 studies [13-22] comprising seven RCTs, two systematic reviews, and one prospective cohort study, with sample sizes ranging from 12 to 201 participants [19, 22]. The RCTs evaluated diverse AI-driven interventions, including nociception-guided analgesia [14, 20], interactive robots for pediatric anxiety [13, 18], and ML for pain prediction [15]. Two systematic reviews synthesized evidence on AI in arthroplasty [16] and surgical outcomes prediction [17], while the cohort study applied ML to cervical radiculopathy recovery [19]. Populations varied from children [13] to adult surgical patients [21], with outcomes spanning pain scores, anxiety, opioid use, and predictive model performance. Study durations ranged from immediate postoperative assessments [15] to 12-month follow-ups [19], reflecting heterogeneous designs and clinical contexts (Table 2).

**Table 2:** A summary of studies included in this study AI: artificial intelligence; RCT: randomized controlled trial; CSA: Children's State Anxiety; NOL™: Nociception Level Index; NRS: Numeric Rating Scale; PACU: post-anesthesia care unit; ML: machine learning; CV-AUC: cross-validated area under the receiver operating characteristic curve; TJA: total joint arthroplasty; LOS: length of stay; PROs: patient-reported outcomes; NDI: Neck Disability Index; EQ5D (QoL): 5-level EQ-5D version (quality of life); ACT:  acceptance and commitment therapy; PROMIS: Patient-Reported Outcomes Measurement Information System; VR: virtual reality

Author (Year)	Country	Study sesign	Sample size	Patient population/Type of surgery	AI approach used	Purpose of AI application	Comparator/ control	Outcomes measured	Follow-up duration	Key findings
Topçu et al. [13] (2023)	Turkey	RCT	84	Children aged between 5 and 10 years undergoing day surgery	Interactive robot (AI-based)	Reduce anxiety, enhance mobilization, and improve parental satisfaction	Standard care during mobilization	CSA score, mobilization duration, parental satisfaction	Postoperative period (unspecified exact duration)	Reduced anxiety before mobilization, increased mobilization duration, and higher parental satisfaction
Fuica et al. [14] (2023)	Israel	Prospective RCT	75	Adult patients undergoing major abdominal surgery	NOL™ – AI-based multiparameter monitoring	NOL-guided intraoperative fentanyl dosing	Standard care – fentanyl based on hemodynamic indices and clinician judgment	Postoperative pain scores (NRS), opioid consumption	180 minutes post-op or until PACU discharge	Significantly lower postoperative pain scores with NOL-guided dosing; no difference in morphine or fentanyl consumption
Morisson et al. [15] (2023)	Israel	Ancillary analysis of RCT (NOLGYN study)	66 analyzed (70 enrolled)	Patients aged between 18 and 75 years undergoing gynecological laparoscopic surgery	ML algorithm (penalized logistic regression)	Predict moderate to severe postoperative pain using intraoperative NOL data	Standard care (non-NOL-guided fentanyl administration)	Postoperative pain (moderate to severe PACU pain)	Immediate postoperative period (PACU)	ML-based model outperformed individual NOL variables with the highest CV-AUC of 0.753
Lopez et al. [16] (2021)	United State	Systematic review	49 studies	Hip and knee arthroplasty (TJA)	AI/ML models	Cognitive support and decision-making (e.g., cost, LOS, complications, pain, PROs)	Not applicable (review study)	Cost, LOS, discharge, readmission, complications, pain, PROs	Varies by study	AI/ML models performed best in predicting complications (AUC 0.84), pain (0.83), and PROs (0.81); lower accuracy for readmission/reoperation (AUC 0.66)
Elfanagely et al. [17] (2021)	United State	Systematic review	45 studies included	Various surgical subspecialties (most common: neurosurgery)	Random forest (n=19), artificial neural networks (n=17), logistic regression (n=17)	Predict surgical outcomes	Conventional statistical models	Postoperative mortality, complications, quality of life, and pain improvement	Not specified	ML models showed improved prediction accuracy compared to conventional methods (measured by AUC)
Lee-Krueger et al. [18] (2021)	Canada	RCT	137	Pediatric patients (4–12 years) undergoing IV line placement before short-stay surgery	Humanoid robot (MEDi®)	Teach deep breathing to reduce pain and fear during IV induction	Standard care with topical anesthetic (Ametop©) only	Pain, fear, IV induction completion	Immediate (before, during, and after IV insertion)	No significant differences in pain/fear; robot group five times more likely to complete IV induction
Liew et al. [19] (2020)	Sweden	Prospective cohort study	201	Individuals with cervical radiculopathy (non-surgical or mixed unclear)	ML methods: LASSO, Boosting, MuARS; traditional: Stepwise regression	Prognostic modeling of clinical outcomes in cervical radiculopathy	Traditional stepwise regression	NDI, EQ5D (QoL), neck pain intensity, arm pain intensity	12 months	All models showed similar performance; MuARS yielded parsimonious models with comparable predictive accuracy
Meijer et al. [20] (2020)	Netherlands	RCT	50	Abdominal surgery under general anesthesia	NOL™ index – multiparameter AI-driven index	Guide intraoperative opioid (fentanyl) dosing	Standard care based on haemodynamics	Postoperative pain score, morphine consumption	PACU	NOL-guided group had significantly lower pain scores (3.2 vs 4.8, P=0.006) despite similar opioid consumption
Anthony et al. [21] (2020)	United State	RCT	82 (76 completed)	Adults undergoing operative fixation for upper/lower extremity fractures	Automated mobile messaging robot	Deliver ACT-based psychological intervention	No-message control group	Opioid use, pain intensity (PROMIS scores)	2 weeks post-op	36.5% reduction in opioid use and lower pain scores in the intervention group
Maani et al. [22] (2011)	United State	Controlled study	12	US soldiers with combat-related burn injuries during wound debridement	Immersive VR	Pain reduction during wound care	Standard of care pharmacologies	Pain intensity (Graphic Rating Scale), pain unpleasantness, time spent thinking about pain, patient satisfaction	Immediate (within session)	Significant pain reduction during VR; greatest effect in patients with the highest initial pain ratings

AI Interventions for Postoperative Pain Management

AI-driven approaches demonstrated significant efficacy in reducing postoperative pain across multiple surgical populations. The Nociception Level Index (NOL™), an AI-based multiparameter monitoring system, was particularly effective in guiding intraoperative fentanyl dosing, leading to lower postoperative pain scores compared to standard care in patients undergoing major abdominal surgery [14]. Similarly, Meijer et al. [20] found that NOL-guided analgesia during abdominal surgery under general anesthesia resulted in significantly reduced pain scores (3.2 vs. 4.8, P=0.006) despite comparable opioid consumption. ML algorithms also showed promise in predicting postoperative pain severity. Morisson et al. [15] developed an ML model using intraoperative nociception data to predict moderate-to-severe postoperative pain with high accuracy (CV-AUC: 0.753), outperforming traditional methods.

Non-pharmacological AI interventions, such as immersive virtual reality (VR), were also effective. Maani et al. [22] reported significant pain reduction during wound debridement in combat-related burn injuries when patients used VR, particularly among those with high baseline pain. These findings suggest that AI can enhance both pharmacological and non-pharmacological pain management strategies.

AI for Reducing Anxiety and Improving Psychological Outcomes

AI-based psychological interventions and robotic assistance were effective in alleviating preoperative and postoperative anxiety. Topçu et al. [13] evaluated an interactive robot in children undergoing day surgery and found reduced anxiety before mobilization, along with increased parental satisfaction. Similarly, Lee-Krueger et al. [18] tested a humanoid robot (MEDi®) to teach deep breathing techniques to pediatric patients before IV line placement. While no significant differences in pain or fear were observed, children in the robot-assisted group were five times more likely to complete IV induction, suggesting improved cooperation.

For psychological outcomes, Anthony et al. [21] implemented an automated mobile messaging robot delivering acceptance and commitment therapy (ACT) to postoperative orthopedic trauma patients. The intervention group showed a 36.5% reduction in opioid use and lower pain intensity scores compared to controls, highlighting the potential of AI-driven behavioral interventions to improve recovery.

AI in Predictive Modeling and Decision Support

ML models demonstrated strong performance in predicting surgical outcomes, though accuracy varied by application. Lopez et al. [16] conducted a systematic review of AI/ML in total joint arthroplasty, finding that models excelled in predicting complications (area under the curve (AUC): 0.84), pain (AUC: 0.83), and patient-reported outcomes (PROs) (AUC: 0.81), but were less accurate for readmission/reoperation (AUC: 0.66). Similarly, Elfanagely et al. [17] reviewed ML applications across surgical subspecialties and noted improved prediction accuracy for mortality, complications, and pain compared to conventional statistical models.

In cervical radiculopathy, Liew et al. [19] used ML algorithms (LASSO, boosting, MuARS) to model postoperative recovery. While all models performed similarly, MuARS provided the most parsimonious solution, demonstrating ML’s utility in prognosticating recovery trajectories.

Quality Assessment Results

Among the seven RCTs assessed using the ROB 2 tool, five studies were rated as having an overall low risk of bias; these included studies by Topçu et al. [13], Fuica et al. [14], Lee-Krueger et al. [18], Meijer et al. [20], and Anthony et al. [21], which demonstrated clear randomization procedures, low levels of missing outcome data, and appropriate handling of reported outcomes. Two studies, Morisson et al. [15] and Maani et al. [22], were rated as having some concerns. In these cases, limitations stemmed from uncertainties in the randomization process or the use of subjective outcome measures such as pain and anxiety, where blinding was either not reported or inadequately addressed (Table 3).

**Table 3:** Assessment for seven randomized controlled trials (RCTs) using the revised Cochrane risk of bias tool for randomized trials (ROB 2) tool

Study	Domain 1: Randomization process	Domain 2: Deviations from the intended interventions	Domain 3: Missing outcome data	Domain 4: Measurement of the outcome	Domain 5: Selection of the reported result	Overall risk of bias
Topçu et al. [13] (2023)	Low	Low	Low	Some concerns	Low	Low
Fuica et al. [14] (2023)	Low	Low	Low	Low	Low	Low
Morisson et al. [15] (2023)	Some concerns	Low	Low	Some concerns	Low	Some concerns
Lee-Krueger et al. [18] (2021)	Low	Low	Low	Some concerns	Low	Low
Meijer et al. [20] (2020)	Low	Low	Low	Low	Low	Low
Anthony et al. [21] (2020)	Low	Low	Low	Low	Low	Low
Maani et al. [22] (2011)	Some concerns	Low	Low	Low	Some concerns	Some concerns

For the three non-randomized studies assessed using the ROBINS-I tool, all were judged to have an overall moderate risk of bias. The prospective cohort study by Liew et al. [19] presented some risk due to a lack of randomization and potential confounding, although outcome measurement and data completeness were adequately addressed. Both systematic reviews, Lopez et al. [16] and Elfanagely et al. [17], exhibited moderate risk primarily due to concerns about outcome measurement and reporting practices. These reviews did not fully describe methods for study selection or data synthesis and did not clearly report how bias was minimized across included evidence, limiting confidence in their conclusions (Table 4).

**Table 4:** Risk Of Bias In Non-randomised Studies - of Interventions (ROBINS-I) assessment for the three non-randomized studies

Study	Bias due to confounding	Bias in the selection of participants	Bias in the classification of interventions	Bias due to deviations from intended interventions	Bias due to missing data	Bias in the measurement of outcomes	Bias in the selection of the reported results	Overall risk of bias
Liew et al. [19] (2020)	Moderate	Low	Low	Low	Low	Low	Low	Moderate
Lopez et al. [16] (2021)	Moderate	Low	Low	Low	Some concerns	Some concerns	Some concerns	Moderate
Elfanagely et al. [17] (2021)	Moderate	Low	Low	Low	Some concerns	Some concerns	Some concerns	Moderate

Discussion

The study highlights the transformative potential of AI-driven approaches in managing postoperative pain, anxiety, and psychological outcomes among surgical patients. The integration of AI technologies, ranging from real-time nociception monitoring to interactive robotics and ML predictive models, demonstrates significant advancements in personalized perioperative care [6]. Notably, AI applications have shown efficacy in reducing subjective distress, optimizing analgesic administration, and improving long-term recovery trajectories. However, the heterogeneity in study designs, patient populations, and outcome measures underscores both the promise and challenges of implementing AI in clinical practice. 

One of the most compelling findings is the role of AI in enhancing postoperative pain management. NOL™, an AI-based multiparameter monitoring system, consistently outperformed conventional hemodynamic-guided analgesia in reducing postoperative pain scores [14, 20]. These results align with emerging evidence that AI-driven nociception monitoring can minimize opioid overuse while improving pain control, a critical consideration in the context of the opioid epidemic [20]. Similarly, ML algorithms demonstrated robust predictive accuracy for postoperative pain severity, with Morisson et al. [15] reporting an AUC of 0.753 for moderate-to-severe pain prediction. This capability to anticipate pain trajectories could enable preemptive interventions, reducing reliance on reactive analgesia. However, the generalizability of these models remains uncertain, as most studies focused on specific surgical populations (e.g., abdominal or gynecological surgeries), limiting broader applicability. 

Beyond pharmacological management, AI-based non-pharmacological interventions, such as VR and interactive robotics, showed promise in alleviating perioperative distress [23, 24]. Maani et al. [22] reported significant pain reduction during wound debridement using VR, particularly in high-pain cohorts, suggesting that immersive technologies may serve as effective adjuncts to traditional analgesia. In pediatric populations, interactive robots reduced preoperative anxiety and improved parental satisfaction [13], while Lee-Krueger et al. [18] found that robot-assisted interventions enhanced procedural compliance, albeit without significant reductions in pain or fear. These mixed outcomes highlight the need for further refinement of non-pharmacological AI tools, particularly in tailoring interventions to specific patient demographics and surgical contexts [25]. 

Psychological outcomes, particularly postoperative opioid use and patient-reported recovery, were also positively influenced by AI-driven interventions. Anthony et al. [21] demonstrated that an automated messaging robot delivering ACT significantly reduced opioid consumption and pain intensity in orthopedic trauma patients. Research indicates behavioral interventions effectively diminish opioid dependence, while studies find new support for this approach [21]. Such trials without blinding procedures cause doubts regarding placebo effects because the evidence clearly demonstrates the requirement for meticulously designed studies to establish isolated benefits from AI therapy [13, 18]. 

The topic of AI-based prediction for surgical outcomes stood as a major point in discussions. Lopez et al. [16], together with Elfanagely et al. [17], performed systematic reviews that demonstrated that ML models produced excellent predictions regarding complications and pain as well as PROs, yet demonstrated weaker performance for readmissions and reoperations. The data implies that AI proves useful in supporting clinical choices, even though its effectiveness varies according to specific situations. Research by Liew et al. [19] proved that ML methods produced equivalent recovery models for cervical radiculopathy as traditional methods, although practical clinical insights were hard to extract from the findings. The mismatch between predictive success and practical system integration needs additional investigation when establishing AI tools for real-time clinical use.

The review showed various issues that restricted the findings across different studies. Two studies with small sample groups [20, 22] and unblinded procedures by researchers [13, 18] appeared frequently as methodological weaknesses throughout existing articles. Additionally, the predominance of short-term follow-ups (e.g., immediate postoperative assessments in Morisson et al. [15]) limits understanding of AI’s long-term impact. The comparison between different AI studies becomes challenging because a heterogeneous combination of AI approaches, including logistic regression along with deep learning, exists. The heterogeneity in AI methodologies-ranging from logistic regression to deep learning-further complicates cross-study comparisons. For example, while Lopez et al. [16] and Elfanagely et al. [17] provided high-level insights into AI’s predictive performance, neither review standardized model reporting, hindering reproducibility. 

The broader literature supports many of these findings while also highlighting gaps. For instance, a meta-analysis by Hashimoto et al. [26] corroborated the efficacy of AI in reducing postoperative pain but noted inconsistent reporting of model validation. Similarly, a scoping review by Lin et al. [27] emphasized the need for patient-centered AI tools, echoing the mixed results seen in pediatric and psychological interventions [13, 21]. These parallels suggest that while AI holds immense potential, its clinical integration requires

Limitations

This review has several limitations. First, the inclusion of heterogeneous study designs (RCTs, cohort studies, systematic reviews) precluded meta-analysis, limiting quantitative synthesis. Second, the predominance of small-scale studies [20, 22] may overestimate effect sizes. Third, the lack of long-term follow-up data in most studies restricts conclusions about sustained AI benefits. Finally, publication bias may favor positive outcomes, as negative or null results are often underreported. 

## Conclusions

AI-driven approaches demonstrate significant potential in improving postoperative pain, anxiety, and psychological outcomes, but their clinical adoption requires addressing key methodological and practical challenges. Future research should prioritize large-scale, blinded RCTs with standardized AI protocols and long-term follow-ups to validate these technologies. By bridging these gaps, AI can transition from a promising tool to a cornerstone of perioperative care. 
